# Potential effects of assisted reproductive technology on telomere length and telomerase activity in human oocytes and early embryos

**DOI:** 10.1186/s13048-023-01211-4

**Published:** 2023-07-04

**Authors:** Betul Tire, Saffet Ozturk

**Affiliations:** Department of Histology and Embryology, Akdeniz University School of Medicine, Campus, 07070 Antalya, Turkey

**Keywords:** Telomere length, Telomerase activity, Oocyte and early embryo quality, Assisted reproductive technology, Biomarker

## Abstract

Telomeres are repetitive DNA sequences at eukaryotic chromosome ends and function in maintaining genome integrity and stability. These unique structures undergo shortening due to various factors including biological aging, consecutive DNA replication, oxidative stress, and genotoxic agents. Shortened telomeres can be lengthened by the enzyme telomerase and alternative lengthening of telomeres in germ cells, early embryos, stem cells, and activated lymphocytes. If telomeres reach to critical length, it may lead to genomic instability, chromosome segregation defects, aneuploidy, and apoptosis. These phenotypes also occur in the oocytes and early embryos, produced using assisted reproductive technologies (ARTs). Thus, a number of studies have examined the potential effects of ART applications such as ovarian stimulation, culture conditions, and cryopreservation procedures on telomeres. Herein, we comprehensively reviewed impacts of these applications on telomere length and telomerase activity in ART-derived oocytes and embryos. Further, we discussed use of these parameters in ART centers as a biomarker in determining oocyte and embryo quality.

## Introduction

Infertility is described as inability to conceive after one year or more of regular unprotected sexual intercourse [1]. As couples postpone childbearing due to socioeconomic conditions, infertility rates have gradually increased in the last decades worldwide [2]. Thus, applications to assisted reproductive technology (ART) centers for having children are progressively increasing. The National Centers for Disease Control and Prevention (CDC) report in 2017 revealed that 1.9% of all live births became as a result of ARTs in the United States [3]. Strikingly, International Committee for Monitoring Assisted Reproductive Technologies (ICMART) reported that to date over 8 million babies were born with ARTs worldwide [4].

It is known that ARTs can lead to short and long term adverse effects such as impaired oocyte maturation, delayed embryonic or fetal development, lower implantation rates, growth retardation, abnormal chromosome segregation, numerical or structural chromosome anomalies, defective genomic imprinting and altered DNA methylation landmarks [5, 6]. Some of these effects such as chromosomal abnormalities are also associated with telomeric changes. After introducing telomere structure, the potential effects of ART applications including ovarian stimulation, culture conditions, and cryopreservation on telomere length and telomerase activity in human oocytes and early embryos were evaluated in the following parts.

### Telomeres: a unique structure at chromosome ends

Telomeres are located at the outermost ends of eukaryotic chromosomes and protect these regions from undergoing degradation so that genome integrity maintains during lifespan. This unique structure was first discovered in the chromosomes of *Drosophila melanogaster* by Hermann Muller in 1938 [7]. The word ‘telomere’ primarily used by Muller consists of the *Greek* words “*telo*” meaning ‘end’, and “*mere*” meaning ‘part’ [7]. In mammals, telomeres comprise evolutionarily conserved tandem repeats of the non-coding sequences (5’-TTAGGG-3’)_n_ [8]. The repetition numbers show variance among species, even among cells and tissues of each individual [9].

**Fig. 1:**
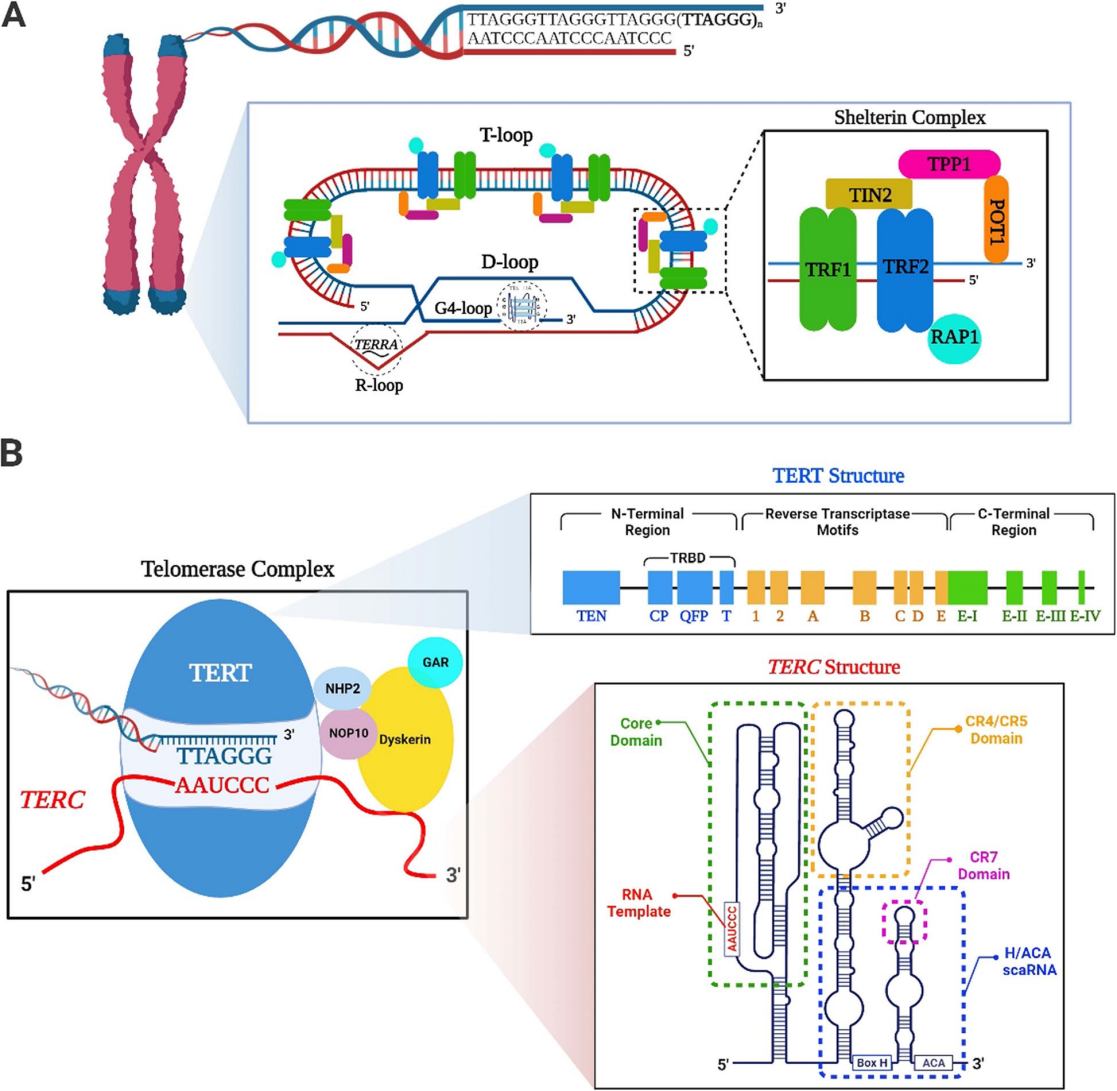
Structure of telomere and telomerase complex. **A **Telomeres are composed of telomeric DNA repeats (5’-TTAGGG-3’)_n_ and shelterin complex that includes TRF1, TRF2, TIN2, TPP1, POT1, and RAP1 proteins. While TRF1 and TRF2 proteins bind to double-stranded DNA, POT1 protein interacts with single-stranded DNA. The 3’ end of telomere known as G-tail folds back to form T- and D-loop through entering into telomeric DNA duplex. The non-canonical secondary DNA structures defined as G4- and R-loop can also be observed in telomeric structure. **B** The telomerase complex is basically formed from TERT and *TERC* subunits. Additionally, the adaptor proteins NHP2, GAR, NOP10, and Dyskerin support structural integrity of this complex. The catalytic subunit TERT contains N- and C-terminal regions and reverse transcriptase motifs between them. These regions harbour subdomains, such as TERT RNA-binding domain (TRBD) at N-terminal region. The *TERC* subunit includes the conserved region (CR) domains, such as the core domain, CR4/CR5, H/ACA box (CR6/CR8) and CR7. This schematic diagram was created using the BioRender Program (BioRenderCompany; Toronto, Canada)

The 3’ single-stranded extension of telomeres includes guanine-rich sequences, referred to as the G-tail or G-overhang. The G-tail not only folds back on itself to form a telomere loop (T-loop) but also invades into double-stranded telomeric DNA to create a displacement loop (D-loop) [10] **(**Fig. 1A**)**. These loops basically contribute telomere elongation process by modulating accessibility of the enzyme telomerase to telomeric site [11]. The closed-loop conformation of telomeres, provided by these loops, preserves chromosome ends from being sensed as double-stranded DNA breaks (DSBs). Thus, DNA damage response activation through classical non-homologous end joining (cNHEJ) and homologous recombination (HR) pathways are repressed [11, 12].

In addition to T- and D-loop formation, telomeres create the non-canonical secondary structures, G4-loop and R-loop [13] **(**Fig. 1A**)**. The G-tail makes extra folds to comprise G4-loop (also known as G-quadruplex) [14]. The basic unit of G4-loop is the G-quartet, which includes four guanine nucleotides. These nucleotides are held together by Hoogsteen hydrogen bonds. Assembly of two or more quartet units promotes G4-loop formation [15]. Importantly, G4-loop participates in suppressing DNA damage signal transduction [16], inhibiting telomerase access [17], protection and stabilization of telomeres [18]. Another secondary structure R-loop is a hybrid of DNA:RNA interaction and plays key roles in regulating *TERRA* (telomeric repeat-containing RNA) gene expression and DNA repair response [19]. Excess R-loop formation on telomeres can cause genomic instability and telomeric replication stress [19]. Notably, the long non-coding RNA *TERRA* is composed of UUAGGG repeats and ubiquitously transcribed by RNA polymerase II from subtelomeric and telomeric regions [20]. *TERRA* functions in protecting chromosome ends, heterochromatinization of telomeres, and inhibiting entrance of telomerase to telomeres [20, 21]. As seen, telomeres are not only composed of repeated sequences also contain exclusive loop structures, which contribute to maintaining telomeric integrity and length in cooperation with *TERRA*.

### The telomere-associated proteins

Some telomeric proteins create a shelterin complex which is composed of the six proteins: telomeric repeat-binding factors 1 (TRF1) and 2 (TRF2), repressor/activator protein 1 (RAP1), protection of telomeres 1 (POT1), TRF1-interacting nuclear protein 2 (TIN2), and TIN2 and POT1 interaction protein 1 (TPP1) [8] **(**Fig. 1A**)**. This complex contributes to maintaining integrity of telomeric DNA, protecting telomeres from DNA damage activation, and regulating telomere lengthening [22, 23].

The basic components of shelterin complex, TRF1 and TRF2 proteins, form a homodimer that directly interacts with the double-stranded telomeric DNA [24]. Consistent with their physical support, loss of TRF1 leads to disrupting localization of shelterin complex and thereby to chromosomal instability [25]. On the other hand, TRF1 is a negative regulator of telomere elongation through blocking access of telomerase to telomeric site by modulating T-loop formation at the 3’ end [26]. Expectedly, while TRF1 overexpression causes a gradual telomere shortening, its dominant-negative mutation stimulates telomere elongation [27]. As a shelterin complex member, TRF2 participates in protecting telomeric ends from DNA damage response activation by promoting T-loop formation [28]. Therefore, conditional deletion of the *Trf2* gene in mouse embryo fibroblasts results in activation of the ataxia telangiectasia mutated (ATM) kinase that induces DNA damage response [29]. In a normal telomere structure, TRF2 hinders telomere lengthening by preventing telomerase access [26]. Upon deficiency of TRF2, end-to-end fusion of chromosomes and growth arrest occur, which may be due to impaired telomere length regulation [30].

Another shelterin complex member POT1 interacts only with the 3’ single-stranded G-tail [24]. POT1 helps protection of telomeres through inducing D-loop formation, and represses the ataxia telangiectasia and Rad3*-*related (ATR) kinase activity that phosphorylates the replication of protein A (RPA) for initiating DNA damage response [31, 32]. On the other hand, POT1 establishes a heterodimer with the TPP1 protein not only to contribute to protecting telomeres but also to modulate telomere length by inhibiting accessibility of telomerase [33]. POT1 also works with TRF2 to regulate telomere lengthening and maintain telomere structure and integrity [31, 34]. As expected, POT1 deficiency results in shortening of G-tail that consequently leads to chromosomal instability, cellular senescence, and apoptosis [31].

The TPP1 protein serves as a bridge between TIN2 and POT1 proteins, and plays a role in sustaining telomere structure through these interactions [35]. The central protein of shelterin complex, TIN2, provides a stable interaction between the TRF1 and TRF2 proteins in addition to TPP1 and POT1 [36, 37]. The essential role of TIN2 is to preserve chromosome ends from unnecessary DNA damage responses via blocking the ATR signaling by the way of establishing an interaction with TPP1 and POT1 proteins [36]. Lacking *Tin2* gene in mice causes embryonic lethality due to severely impaired telomeric integrity [38]. The RAP1 protein hinders cNHEJ repair activation via capping telomeres, and thus prevents end-to-end fusion among chromosomes [39]. A recent study revealed that RAP1 also contributes to preserving telomeres in the human cells, undergoing replicative senescence [40]. RAP1 deficiency leads to telomere shortening and increase of DNA damage levels, especially DSBs [41].

Overall, certain telomere-associated proteins comprise the shelterin complex that carries out two important missions: protection of telomeres from shortening through modulating telomerase accessibility and prevention of telomeres from sensing as DNA breaks. Loss of these proteins leads to increased apoptosis, cellular senescence, and genomic instability due to excessive telomere shortening.

### Telomere shortening and lengthening

**Fig. 2:**
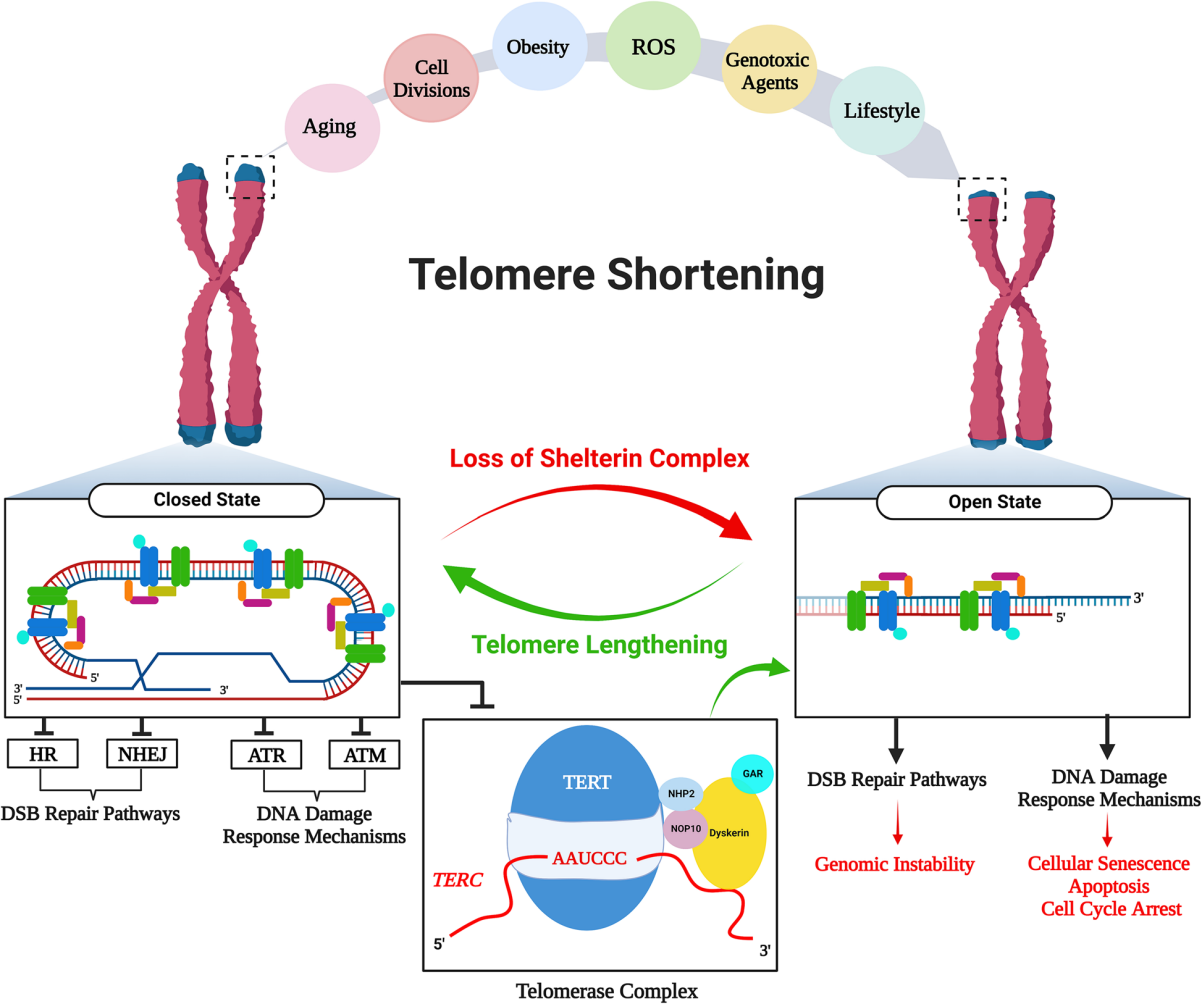
Telomere shortening: causes and outcomes. Telomeres can shorten due to various factors such as biological aging, cell divisions, obesity, reactive oxygen species (ROS), genotoxic agents, and lifestyle. In this case, telomeres may switch from a closed state to an open state. In the closed state showing normal telomeric structure, not only the double-stranded break (DSB) repair pathways, homologous recombination (HR) and non-homologous end joining (NHEJ), but also DNA damage response mechanism by the actions of ATR and ATM are repressed to prevent telomeres from being sensed as DSBs. In the open state that may appear owing to excessive shortening of telomeres, because of activating the DSB pathways, the possible outcomes involving genomic instability, cellular senescence, apoptosis, and cell cycle arrest may occur. Once short telomeres are elongated to normal lengths by the enzyme telomerase or alternative lengthening of telomeres (ALT) mechanism, cellular functions can be reestablished. This schematic diagram was created using the BioRender Program (BioRenderCompany; Toronto, Canada)

In eukaryotic cells, telomeres can be found in the two states: open or closed **(**Fig. 2**)**. Telomere shortening and functional loss of the telomere-associated proteins such as POT1 and TRF2 bring telomeres into an open state [12, 42]. In this state, ‎ATM- and ATR-dependent DNA damage response pathways, including cNHEJ and HR, are activated. This may result in genomic instability, cellular senescence, apoptosis, and cell cycle arrest [12, 18]. The closed state is basically established by the telomere-associated proteins, loops and secondary structures. Indeed, this state can be seen only if telomeres are within a length of normal ranges.

A great number of factors such as incomplete DNA replication, consecutive cell divisions, biological aging, lifestyle, reactive oxygen species (ROS), genotoxic agents, and ART applications may cause a gradual decrease in telomere length [24, 43] **(**Fig. 2**)**. As is well-known, short telomeres can be elongated by the two main mechanisms: telomerase-based and alternative lengthening of telomeres (ALT) in the activated lymphocytes, germ cells, early embryos, and stem cells [44, 45]. One or both of these mechanisms are used dependent on cell type, cell cycle phase, and inducing intracellular signaling.

The enzyme telomerase is in a structure of ribonucleoprotein, responsible for elongating telomeres by adding the telomeric repeats onto chromosome ends. Basically, telomerase is composed of two main components: telomerase reverse transcriptase (TERT) and telomerase RNA component (*TERC*) **(**Fig. 1B**)**. The *TERC* RNA consists of 451 nucleotides in humans and serves as a template for telomeric repeat synthesis. It contains conserved region (CR) domains such as core domain, CR4/CR5, H/ACA box (also known as CR6/CR8) and CR7, which together play an essential role in interacting with TERT [46] **(**Fig. 1B**)**. The H/ACA box also contributes stability of the formed telomerase complex [46, 47]. Consistent with these basic functions, *Terc*-deficient mice exhibited telomere shortening, aneuploidy, and end-to-end fusion among chromosomes in the mouse embryonic fibroblasts [48] as well as defective fertilization and cleavage in the early embryos [49].

The catalytic subunit TERT includes the three main regions: N-terminal extension (NTE), central catalytic reverse transcriptase (RT), and C-terminal extension (CTE) [50, 51] **(**Fig. 1B**)**. The NTE region contains two domains: the telomerase essential N-terminal domain (TEN) that implicates in recognizing DNA sequence and telomeric elongation, and the telomerase RNA-binding domain (TRBD) that contributes to binding to *TERC* subunit [51]. The RT region involves a catalytic unit, which provides the enzymatic activity. While *TERC* gene is ubiquitously expressed in most tissues independent from presence of telomerase activity [52], *TERT* gene is exclusively expressed in the cells having telomerase activity. In other words, there is a strong correlation between TERT level and telomerase activity whereas *TERC* profile is accepted as a rate limiting factor for telomerase activity [53]. Therefore, telomerase-based telomere elongation takes place only in the TERT-expressing cells, such as germline cells, granulosa cells, endothelial cells, certain types of stem cell population, cancer cells, activated lymphocytes, and early embryos; however, no telomerase activity was noted in many somatic cells [54–56].

In addition to basic function of TERT in telomere lengthening, it also exhibits non-canonical roles in modulating gene expression, signal transduction, stress response, and mitochondrial metabolism [57]. A recent study has demonstrated that under stress conditions, TERT is undergone phosphorylation by the SRC kinase and then transported into mitochondria to protect from increased oxidative stress [58]. Moreover, TERT participates in regulating expression of the WNT/β-catenin signaling-target genes by acting as a transcription (co-)factor [59]. Thus, deficiency of TERT in mice led to decreased survival and restricted tissue renewal owing to excessively shortened telomeres and loss of the non-canonical functions [60].

#### Alternative telomere lengthening

Telomeres can also be elongated with the ALT mechanism regardless of telomerase activity. It relies on homologous recombination process between telomeres of sister chromatids [61]. This mechanism is commonly used in the cell types showing heterogeneous telomere lengths, telomere sister chromatid exchanges, many extrachromosomal telomere repeats and ALT-associated promyelocytic leukaemia (PML) bodies (APBs) [61, 62]. APBs harbor telomeric DNA sequences, several shelterin complex components (e.g., TRF1 and TRF2), and DNA recombination helicases like WRN and BLM [63, 64]. Importantly, ALT mechanism is mainly modulated by the epigenetic changes such as DNA methylation landmarks on telomeres [65, 66]. Nevertheless, further studies are required to elucidate the intracellular signaling pathways or factors regulating this process.

### Telomere length and telomerase activity in human oocytes and embryos

**Fig. 3:**
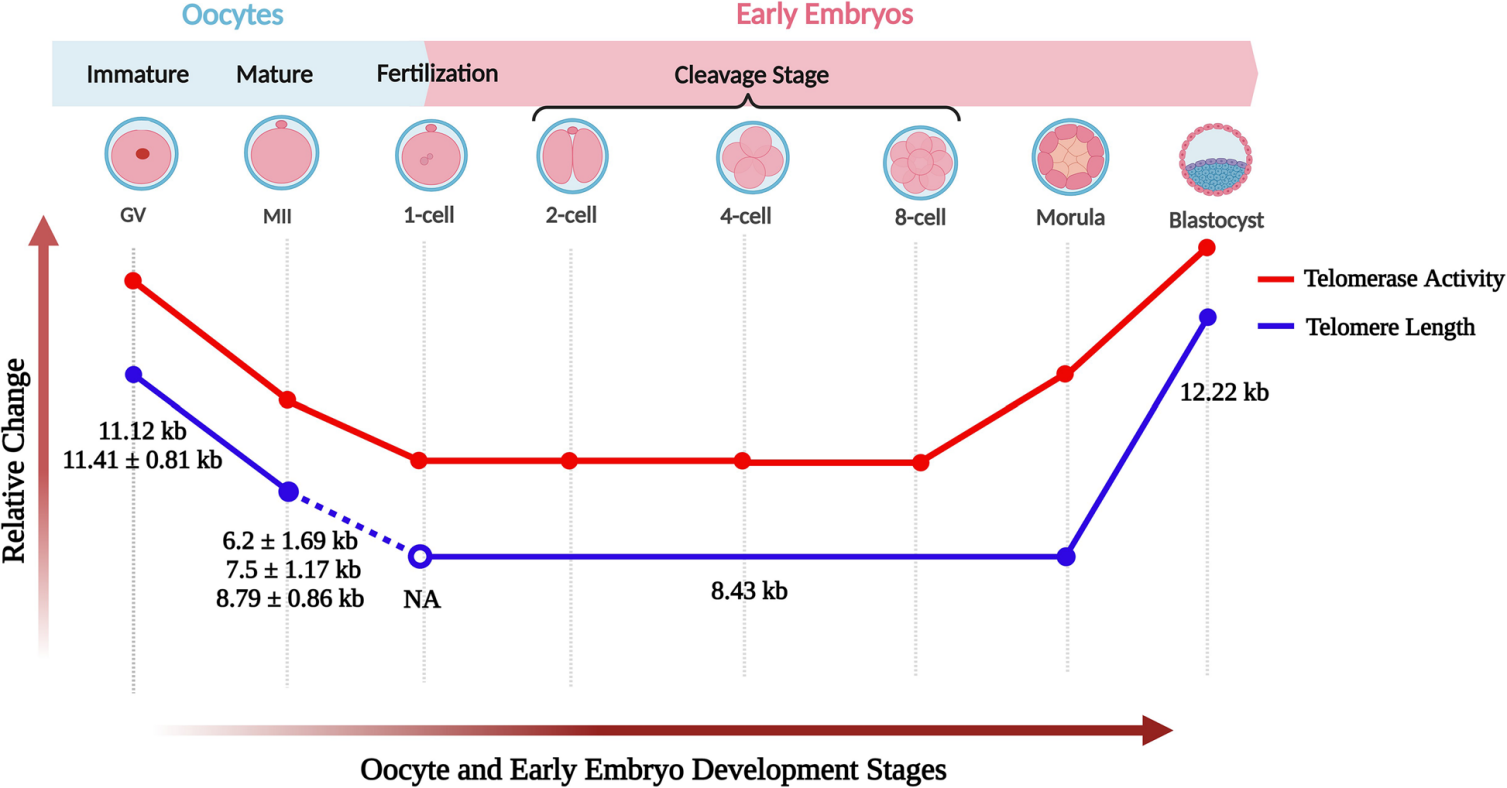
Telomere length and telomerase activity in human oocytes and early embryos. Both parameters show similar relative changes in the oocytes from germinal vesicle to metaphase II (MII) stages and in the early embryos from 1-cell to blastocyst stages. Telomere length and telomerase activity level decreased from GV oocytes to 1-cell embryos, remained stable from 1-cell to 8-cell/morula stage embryos and remarkably increased in blastocysts. The exact telomere length(s) (mean ± SD/SEM) of each oocyte and embryo stage is given below as a blue line. It is important to note that the telomere length of 8.43 kb was determined in the pool of cleavage stage embryos. NA, not analyzed; kb, kilo base pairs. This schematic diagram was created using the BioRender Program (BioRenderCompany; Toronto, Canada)

Telomere length and telomerase activity show dynamic changes during oocyte maturation and early embryo development in humans (reviewed by our group [44]). Due to ethical concerns, a limited number of studies have measured telomeres and telomerase activity in the oocytes and early embryos at different stages. Telomerase activity was detected in human fetal, newborn and adult ovaries as well as in blastocysts, but no activity was noted in unfertilized oocytes using telomere repeat amplification protocol (TRAP) [54]. Subsequently, the same group also demonstrated that human immature oocytes had higher telomerase activity than mature ones [67]. In early embryos, telomerase activity at low levels from 1-cell to 8-16-cell stages progressively increased from morula to blastocysts [67] **(**Fig. 3**)**. These findings suggest that telomerase activity is kept at high levels before initiating oocyte maturation and embryo implantation possibly for the purpose of increasing telomere length.

When telomere length was measured by quantitative fluorescence in situ hybridization (Q-FISH), the germinal vesicle (GV) oocytes of women who underwent ART had a telomere length of 11.12 kb [68]. On the other hand, Keefe and colleagues found the mean telomere length of human mature oocytes as 7.5 + 1.17 kb [69]. In a later work in which telomeres were measured with Q-FISH, human immature oocytes (11.41 ± 0.81 kb) possessed significantly longer telomeres than that of the mature oocytes (8.79 ± 0.86 kb) [70]. Overall, immature oocytes have longer telomeres when compared to mature ones **(**Fig. 3**)**, which possibly arises from presence of higher telomerase activity in immature oocytes. As it is impossible to measure telomeres of oocytes that will be used in fertilization, their polar bodies seem to be a reasonable way of predicting oocyte telomere length. In fact, human MII oocytes and their polar bodies were found to have similar telomere lengths, which was analyzed using single-cell telomere length measurement method [71]. In human early embryos, consistent with their telomerase activity profiles, telomere length was 8.43 kb in the cleavage-stage embryos (including 2-4-cell and 5-8-cell embryos) and reached to 12.22 kb in blastocysts [68]. However, telomere length of each embryonic stage and potential correlation between telomere length of polar bodies and developing embryos remain elusive.

### Effects of ART on telomere and telomerase activity

ART includes a number of procedures to achieve pregnancy. The first successful ART application through using in vitro fertilization (IVF) was the birth of Louise Brown in 1978 [72]. After that many children were born and their numbers have progressively increased in the last decades. As couples delay childbearing due to socioeconomic reasons [73], ART gains importance. Advancing maternal age is associated with enhanced aneuploidy rates, impaired meiotic spindles, abnormal cohesin formation, and missegregation of chromosomes in the oocytes and early embryos [74, 75]. Therefore, infertility rates gradually increase among couples. Since telomeres play key roles in various biological events such as meiotic recombination, maintenance of genomic integrity, and chromosome segregation [76], their erosion may lead to decrease of oocyte quality and developmental failures [77, 78].

The basic steps of ART application are ovarian stimulation, in vitro culture, cryopreservation, and embryo transfer. During these procedures, oocytes and embryos are exposed to distinct chemicals and factors, involving gonadotropins, oxygen, carbon dioxide, changed ovarian microenvironment, growth factors, and various metabolites [79, 80]. They may directly or indirectly influence intracellular events. In the following sections, we reviewed possible effects of these ART procedures on telomere length and telomerase activity, which may eventually impact oocyte and embryo quality.

#### Ovarian stimulation

Controlled ovarian stimulation is a protocol by which external hormone administration contributes to obtaining more oocytes from women applied to ART centers or from experimental animals. For this purpose, recombinant follicle-stimulating hormone (rFSH), human menopausal gonadotropin (hMG), human chorionic gonadotropin (hCG), gonadotropin-releasing hormone (GnRH) analogues, estrogen inhibitors (such as clomiphene citrate) or aromatase inhibitors (for example, Letrozole) are being used to stimulate follicular development and subsequently ovulation [81]. The GnRH analogues acting as GnRH agonist or antagonist are employed to prevent endogenous FSH and LH production, which may result in premature follicle and oocyte maturation [82]. As GnRH agonists display a high affinity to GnRH receptors in the pituitary gland, gonadotropin production is largely suppressed. On the other hand, GnRH antagonists bind to the GnRH receptors by competing with endogenous GnRH to block gonadotropin secretion from pituitary gland. While aromatase inhibitors hinder estrogen synthesis in the granulosa cells using androgen precursors, estrogen inhibitors prevent binding of endogenously produced estrogen to its receptors [83]. As a result, distinct ovarian stimulation protocols using different chemicals at variable doses are being utilized in ART centers and animal studies. Importantly, several studies highlighted that individualized stimulation protocols according to patient’s age, body mass index (BMI), antral follicle count (AFC), and anti-Mullerian hormone (AMH) levels should be used to increase the efficiency, and even quality of obtained oocytes [84–86].

In vitro and in vivo studies on experimental animals revealed that ovarian stimulation leads to decreased oocyte and embryo quality, low implantation rates, delayed prenatal and postnatal development (reviewed in [87]). Additionally, increased rates of postimplantation mortality and morphologically abnormal early embryos with fragmented blastomeres, and defective cleavage were observed [88–90]. A recent study on mice reported that a high dose of equine chorionic gonadotropin (eCG) administration causes aneuploidy in the IVF-derived embryos possibly owing to elevated oxidative stress [91]. On the other hand, mild stimulation regimens in women decreased the number of aneuploid embryos [92]. These findings indicate that gonadotropins are associated with a number of defective changes during early and late development periods. However, it remains elusive whether all gonadotropins elucidate these phenotypes by acting on the same mechanisms.

**Fig. 4:**
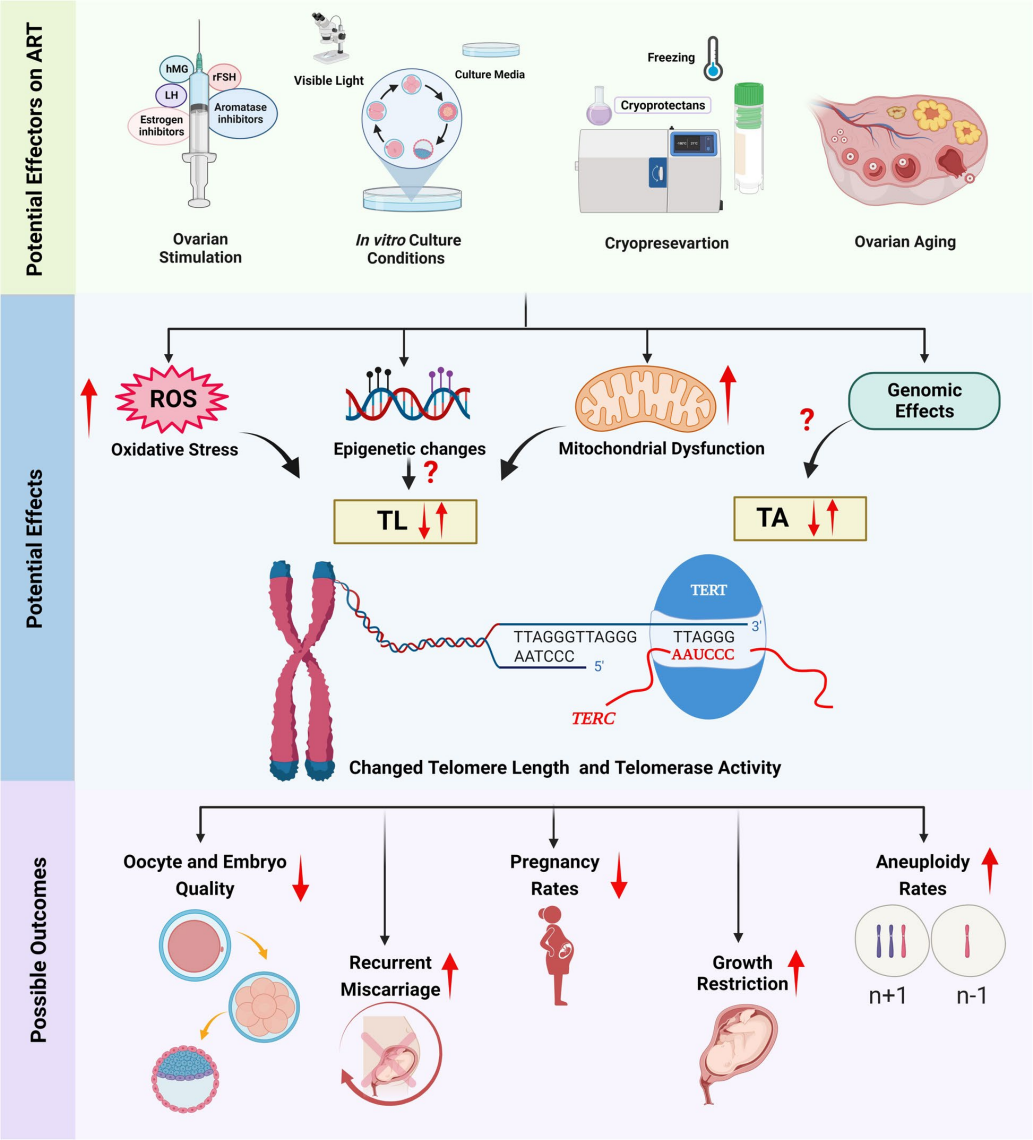
Potential effects of the assisted reproductive technology (ART) applications and ovarian aging on reproductive features. Ovarian stimulation, in vitro culture conditions, cryopreservation, and ovarian aging enable to change telomere length (TL) and telomerase activity (TA) trough increasing oxidative stress, epigenetic changes, mitochondrial dysfunction, and genomic effects (by the way of regulating *Tert* gene expression). As a consequence of these effects, while oocyte and embryo quality as well as pregnancy rates decrease, the other features involving recurrent miscarriage, growth restriction, and aneuploidy rates increase. This schematic diagram was created using the BioRender Program (BioRenderCompany; Toronto, Canada)

The pregnant mare serum gonadotropin (PMSG), commonly used to stimulate follicular development, at 10 IU/mL enabled to increase the levels of *Tert* mRNA, TERT protein and telomerase activity in rat granulosa cells [93]. Increased TERT protein levels contributed to enhanced steroidogenic gene expression, such as *STAR*, *CYP11A*, and *HSD3B* as well as estrogen and progesterone concentrations in the rat and human granulosa cells. Consistent with these findings on granulosa cells, in vivo- and in vitro-produced mouse blastocysts following superovulation had longer telomeres than that of in vivo-produced blastocysts from natural mating cycles [94]. As ovarian stimulation leads to inducing *Tert* gene expression by the action of estrogen on its promoter [95] and increasing oxidative stress through making mitochondrial DNA damage [96], elevated telomerase activity may result in elongating telomeres during early embryo development periods **(**Fig. 4**)**. Also, TERT protein may be increased upon ovarian stimulation to perform its non-canonical roles, including non-telomeric DNA damage response, stimulation of cell growth and proliferation, and attenuation of mitochondrial dysfunction due to oxidative stress [97].

Several studies reported that superovulation can cause alterations in the epigenome of oocytes and embryos [87], especially on global DNA methylation [98, 99] and DNA imprinting profiles [6, 100]. Further evidence has been provided by Yu et al. (2019) that superovulation changes genome-wide methylation in mouse early preimplantation embryos [99]. As telomere length reprogramming in oocytes and embryos may be related to methylation and demethylation dynamics [101], telomere changes in superovulated oocytes and embryos should also be explored in the aspects of established DNA methylation patterns.

To the best of our knowledge, no study examined possible effects of ovarian stimulation protocols on the telomeres of human oocytes and early embryos. In the leukocytes obtained from women with dyskeratosis congenita (DKC), telomere length was found to be increased as a result of ovarian stimulation treatment [102]. The elevated estrogen levels due to controlled ovarian stimulation may lead to increased TERT expression, and thereby telomerase activity in these cells. Further studies are needed to evaluate how ovarian stimulation can influence telomere length in the human oocytes and embryos. Another important issue is that repeated ovarian stimulation is generally used in ART centers. Potential effects of consecutive gonadotropins should be evaluated in the human oocytes and early embryos at least in the sibling cells such as polar bodies, and cumulus cells as well as in biopsied embryonic cells. Given that increased oxidative stress and mitochondrial DNA damage upon repetitive superovulation [96], it would be important to evaluate TERT levels in these cells not only for its primary role in telomere lengthening but also for its non-conical functions.

#### In vitro culture

Mature oocytes obtained following ovarian stimulation and retrieval are fertilized with competent sperm and then cultured in vitro to produce early embryos. These embryos are transferred according to legal permission for the purpose of achieving a healthy pregnancy. During these processes, oocytes and embryos are exposed to various in vitro culture conditions such as culture media, oxygen levels, temperature, osmolality, and pH, which may increase ROS amounts [103, 104] (Fig. 4).

An interesting study by Oh et al. (2007) reported that subjecting hamster embryos to light in the blue range of visible spectrum (445–500 nm) caused a reduction in blastocyst quality and formation, increase of blastomere apoptosis, and excess ROS generation in morulae, whereas red ray provided the best embryonic development [105]. In addition to red light and blue light, enhanced Fe^2+^ and Cu^2+^ ion profiles in culture medium contribute to increased oxidative stress [106]. Therefore, choosing the culture medium containing antioxidants seems to be reasonable to prevent adverse effects of oxidative stress. As is known, increased oxidative stress not only entails decrease of telomere length but also leads to mitochondrial dysfunction.

A recently published study demonstrated that in vitro culture does not change telomere length in blastocysts [94]. Another study similarly reported that in vitro culture did not affect telomere length in blastocysts, as in mouse embryos (E3) at the late cleavage stage [107]. These results suggest that in vitro culturing does not seem to influence telomere length of late preimplantation embryos. Surprisingly, prolonged culture of IVF-derived blastocysts (120 h versus 96 h) led to increase of telomere length [94]. Mild oxidative stress, produced during embryo culturing, may have triggered telomere lengthening [108] via altering DNA methylation pattern. On the other hand, in vitro culturing was discovered to suppress telomerase activity in the early mouse blastocysts at E3.5 [107]. This may arise from potential effects of culture conditions on DNA methylation state and transcription factor binding to upstream of the *Tert* gene, which might result in decreased telomerase activity. With regarding this insight, Anifandis et al. (2014) showed that extended embryo culture can cause aberrant DNA methylation establishment [109], which may be associated with changed telomere length in the early embryos [101]. Nevertheless, it is unknown how telomeric lengthening takes place in in vitro-cultured embryos without utilizing telomerase.

Overall, in vitro culture is an indispensable ART process and includes a wide range of factors that may influence intracellular events toward telomere length regulation. However, there are a limited number of studies that examined potential impacts of these factors on telomeres and the related parameters such as telomerase activity and telomere associated protein levels. Thus, further studies are required to evaluate short and long term effects of in vitro culture conditions on telomeres.

#### Cryopreservation

Cryopreservation is a protocol for short- or long-term preservation of cells and tissues without changing their structural features [110]. It is frequently utilized to store oocytes and embryos, which are obtained from women who underwent ART applications or would be treated with chemotherapy for some diseases such as cancer [111].

Cryopreserved cells are inevitably exposed to increased oxidative stress as a result of using some cryoprotectants, including dimethyl sulfoxide (DMSO), glycerol, and 1, 2-propanediol, etc. [112]. Honda et al. (2001) found that cryopreservation triggers replicative senescence and single-stranded DNA breaks in the telomeric DNA of retinal pigment epithelial cells, which may result in telomeric shortening [113]. A recently published study revealed that telomere length of human ovarian tissues cryopreserved by slow-freezing or thawing methods (8.34 ± 1.83 kb) predominantly decreased when compared to the fresh ones (9.57 ± 1.47 kb) [114]. As is seen, cryopreservation has an adverse effect on telomeres most likely due to increased ROS levels. Determining potential effects of cryopreservation protocols on telomere length and telomerase activity in human oocytes and embryos merits further investigation since these protocols gain more importance to protect reproductive life of patients with deleterious diseases in the early adult periods.

#### Ovarian aging

Because of delaying childbearing in women in the last decades, potential effects of reproductive aging especially ovarian aging on the telomeres of oocytes and early embryos gain increasing importance. As is known, telomere length is a key factor correlating with reproductive lifespan as well as life expectancy [115]. Consistently, leukocytes from women in postmenopausal period had shorter telomeres than those at similar ages or still menstruating [116]. Indeed, women having longer telomeres in their leukocytes enter menopause approximately three years later when compared to the ones with shorter telomeres [116]. Long telomeres in leukocytes were also found to be related to extending fertility feature in aging women [117]. These findings suggest that telomere length in leukocytes have a potential in giving information regarding to reproductive lifespan of aging women.

Telomere shortening in oocytes can lead to decreased synapsis and chiasmata formation, enhanced embryo fragmentation, cell cycle arrest, dysmorphology in meiotic spindles, and chromosomal abnormalities [115]. The most prominent factors entailing telomere attrition are increase of ROS levels and mitochondrial DNA damages during cellular aging in human body [118]. As a result, exposure to aged ovarian microenvironment that generally accompanies with oocyte aging is closely associated with telomere shortening [119, 120], and therefore developmental competency of these oocytes gradually decrease [77, 121]. As telomere shortening was found to be related to recurrent miscarriage, aneuploidy, ovarian insufficiency, growth restriction, and declined ART success [121, 122] **(**Fig. 4**)**, potential biomarkers that give exact information on telomere length of aging oocytes should be discovered. First and secondary polar bodies seem to be the best reasonable samples to reach telomere length of sibling oocytes. If telomere attrition plays an important role in oocyte aging, then activating telomerase might prove a useful strategy to rejuvenate ovaries [123]. However, most key cellular determinants of oocyte aging, e.g., chromosome chiasmata, synapsis and cohesions, are laid down during fetal oogenesis, making telomerase activation unlikely to benefit oocytes in women [123].

### Measuring telomere length and telomerase activity for predicting oocyte and embryo quality

In ART centers, choosing competent oocytes and transfer of high-quality embryos are very important to achieve successful live births. As mentioned in the previous studies [124, 125], morphological criteria seem to be insufficient to define oocytes and early embryos with high quality. More reliable molecular biological markers such as measuring telomere length and telomerase activity are required to predict oocytes and embryos with high quality.

With regard to this subject, Keefe et al. (2005) reported that telomere length in the oocytes from women who underwent IVF is negatively correlated with cytoplasmic fragmentation in day 3 embryos [126]. In more detailed, early embryos with long telomeres seem to be more resistant to attrition, while embryos with short telomeres develop some cellular senescence phenotypes, including cytoplasmic fragmentation, cell cycle arrest, and death. It was also demonstrated that telomere shortening increases the possibility of aneuploidy occurrence in human oocytes and embryos [127]. Consistent with these findings, a research on sister oocytes revealed that there is a close relationship between telomere length and successful pregnancy [69]. The mean telomere length of sister oocytes from women who became pregnant (7.5 ± 1.17 kb) was longer than sister oocytes from women who did not conceive (6.2 ± 1.69 kb) [69] **(**Fig. 3**)**. By contrast, Turner and colleagues indicated that telomere length in oocytes, spermatozoa, and early embryos cannot be confidently utilized to predict fertility features and clinical pregnancy outcomes because there may appear predominant changes during the process of meiotic or mitotic recombination [70]. A potential explanation for the discrepancy in these results is that Turner et al. (2013) employed Q-FISH to measure telomeres in cleavage state embryos, a method that has been validated only in metaphase-arrested cells, not in dividing cells [70]. Despite presence of opposite insights on this issue, testing telomere length may predict developmental features, at least for oocytes **(**Table 1**)**.

**Table 1:** Predicted reproductive outcomes upon assisted reproductive technology (ART) treatment by measuring telomere length and telomerase activity in human oocyte, polar body, blastomere, cumulus cells, granulosa cells, and luteinized granulosa cells. IVF, in vitro fertilization; qPCR, quantitative polymerase chain reaction; Q-FISH, quantitative fluorescence in situ hybridization; TRAP, telomere repeat amplification protocol; TRF, telomere restriction fragment

Analyzed sample	Parameter	Method	Predicted outcomes	References	
**Oocyte**	Telomere length **↓**	Q-FISH	➢ Cytoplasmic fragmentation in day 3 embryo **↑** ➢ Apoptosis in day 3 embryo **↑**		Keefe et al. 2005 [126]
		FISH	➢ Pregnancy rate **↓**		Keefe et al. 2007 [69]
**Polar body**		qPCR	➢ Aneuploidy rate **↑**		Treff et al. 2011 [127]
**Blastomere**					
**Cumulus cells**	Telomere length **↑**	qPCR	➢ Rate of competent oocytes **↑** ➢ Embryo quality proportion **↑**		Cheng et al. 2013 [128]
**Lymphocytes**	Telomere length **↓**	qPCR	➢ Number of aneuploid embryos **↑**		Hanson et al. 2021 [122]
			➢ Idiopathic recurrent pregnancy loss **↑**		Thilagavathi et al. 2013 [129]
			➢ Fertility problems **↑** ➢ IVF application rate **↑**		Czamanski-Cohen et al. 2015 [130]
	Telomere length **↑**	TRF	➢ Oocyte quality rate **↑**		Michaeli et al. 2022 [117]
**Granulosa cells**	Telomere length **↑**	qPCR	➢ Aneuploidy rate **↓**		Yu et al*. *2022 [131]
	Telomerase activity **↑**	TRAP	➢ Pregnancy rate **↑**		Wang et al. 2014 [132]
**Luteinized granulosa cells**		Mathematical model	➢ Clinical outcomes of ART treatment **↑**		Portillo et al. 2019 [133]

The main challenge herein is that how telomere length can be measured in oocytes without affecting their developmental progression to embryos. The first polar body, also defined as sister of an oocyte, may be used in measuring telomeres [77]. Indeed, telomere length of polar bodies contributes to anticipating fragmentation status of human embryos [134] and aneuploidy possibility in sibling oocytes [127]. Measuring telomere length in biopsied blastomeres and trophectoderm cells may provide to predict developmental potential of the resulting embryos. In the last decades, cumulus cells, granulosa cells, and leukocytes have been considered as alternative sources in detecting telomere length for the purpose of predicting oocyte and embryo potential.

As is known, cumulus cells surrounding oocytes contribute to regulating maturation through providing nutrient supply and transferring some special factors via gap junctions [135]. Thus, analyzing some parameters e.i., telomere length in these cells may help to obtain information related to development capacity of oocyte. Indeed, relative telomere length of cumulus cells surrounding immature oocytes was shorter than that of cumulus cells around mature oocytes [128]. However, a recently published study reported that telomere length of cumulus cells retrieved on the day of oocyte pick-up (OPU) was not associated with patient’s age or whole chromosome aneuploidy in women undergoing ART [122]. As a result, measuring telomere length in cumulus cells may be utilized as a biomarker for predicting oocyte quality, but not for chromosomal aneuploidy.

Several studies have further focused on whether telomere length in leukocytes can be used as a biomarker for predicting oocyte quality or other reproductive properties **(**Table 1**)**. Xu et al. (2017) reported that women with ovarian failure possessed shorter telomere length in their peripheral blood leukocytes compared to control ones [136]. Similarly, lymphocytes of the couples experiencing idiopathic recurrent pregnancy loss had shorter telomeres [129]. Lymphocytes of women undergoing ART application also exhibited shorter telomere length when compared to healthy women, who did not apply [130, 137]. Moreover, there was a correlation between short telomeres in leukocytes and higher rate of embryonic aneuploidy among ART-applied women [122]. A recent study by Wang et al. (2022) reported that leukocyte telomere length of one-year-old children conceived by ART was shorter than those conceived spontaneously [107]. Further, there was an association between blastocyst-stage embryo transfer and shorter leukocyte telomere length in ART-conceived children.

On the other hand, there were opposite findings related to leukocyte telomere length and reproductive features. For example, a recent study revealed that IVF success is not associated with telomere length in leukocytes [137]. Further, Yu et al. (2022) demonstrated that telomere length in leukocytes cannot be used to predict aneuploidy rate as an alternative to preimplantation genetic test for aneuploidy (PGT-A) cycles [131]. Importantly, telomere length of lymphocytes does not correlate with those of oocytes, granulosa and cumulus cells [131, 138, 139], most likely because leukocytes are differently exposed to various factors, such as hormones, aging, and consecutive cell divisions [140]. Consequently, controversial findings consider us that telomere length results of leukocytes should be carefully evaluated to predict developmental potentiality of oocytes and embryos. Further studies are also required to understand how changed telomere length in leukocytes can affect reproductive features.

Based on the literature analysis, only one recent study evaluated telomere length in granulosa cells from infertile patients and reported that it can be used as a predictor of aneuploidy rate in young women [131]. On the other hand, Wang et al. (2014) demonstrated that telomerase activity may be a more specific determinant in predicting reproductive features [132] **(**Table 1**)**. Women with higher telomerase activity in luteinized granulosa cells exhibited higher pregnancy rates when compared to women with normal telomerase activity levels, whereas no difference was noted for telomere length among these groups [132]. In parallel with that Portillo et al. (2019) revealed that measuring telomerase activity in luteinized granulosa cells may contribute to predicting clinical outcomes of ART treatment [133]. It remains elusive how changed telomerase activity and telomere length in cumulus and granulosa lutein cells influence pregnancy outcomes.

Overall, although it is not possible to directly measure telomere length and telomerase activity in oocytes selected for ART application, the oocytes and embryos with high quality may be predicted through measuring these parameters in cumulus cells, granulosa lutein cells, polar bodies, and leukocytes. Improving more sophisticated and rapid technologies with a high accuracy to measure telomere length and telomerase activity would contribute to increasing ART success rates.

## Conclusion

Telomere length and telomerase activity exhibit dynamic changes during oocyte maturation and early embryo development. Accumulating evidence suggests that measuring telomere length and telomerase activity in polar body, cumulus cells, granulosa cells, and leukocytes can contribute to predicting developmental potential of oocytes and embryos, which may help to increases ART success rates. As available technology does not give a chance to measure telomere length and telomerase activity in the oocytes that will be used in ART applications, measuring these parameters by indirect ways seems to be the only option. During ART procedures, oocytes and embryos are exposed to many environmental factors and chemicals, which can adversely affect telomeres and telomerase activity. These effects should also be kept in mind while making a prediction on oocyte and embryo quality. In this context, future studies should focus on by which mechanisms ART procedures change telomere length and telomerase activity in human oocytes and early embryos as well as short and long term effects of these changes on embryonic and fetal development.

## Data Availability

Not applicable.
